# Traditional Chinese Medicine for Diabetic Sarcopenia: A Review and Its Related Mechanisms

**DOI:** 10.1155/jdr/6655697

**Published:** 2026-07-01

**Authors:** Zicheng Ye, Haoyu Yuan, Hanyu He, Weibo Wen

**Affiliations:** ^1^ Yunnan University of Chinese Medicine, Kunming, China, ynutcm.edu.cn

**Keywords:** diabetic sarcopenia, sarcopenia, traditional Chinese medicine

## Abstract

As societies age worldwide, diabetic sarcopenia has become increasingly common. The development of this disorder involves intricate pathophysiological processes, with contributions from multiple mechanisms: insulin resistance, ongoing inflammatory responses, oxidative damage, buildup of advanced glycation end products (AGEs), compromised mitochondrial function, and alterations in gut microbial composition. The present review comprehensively analyzes the epidemiological patterns and pathological processes associated with diabetic sarcopenia, with special attention to the therapeutic benefits and mechanistic insights of traditional Chinese medicine (TCM). Rooted in substantial clinical experience, TCM implements multitargeted therapeutic approaches using both classical compound formulas (e.g., Sijunzi decoction, Buzhong Yiqi decoction, Bazhen decoction, and Shenling Baizhu powder) and purified bioactive constituents from individual herbs (including astragalus polysaccharide, puerarin, *Lycium barbarum* extract, and magnesium tanshinate). The therapeutic effects encompass optimization of glucose metabolism, stimulation of muscle protein synthesis, inhibition of proteolysis, and reduction of inflammatory and oxidative damage—demonstrating the holistic TCM advantage of “co‐treatment of glucose metabolism and muscle function.” This work provides scientific rationale and clinical evidence to support TCM‐based strategies for preventing and treating diabetic sarcopenia.

## 1. Introduction

Type 2 diabetes mellitus (T2DM) constitutes a frequently encountered chronic metabolic pathology, distinguished by insulin resistance (IR) and sustained hyperglycemia as its cardinal features [1]. The upward trajectory in T2DM prevalence contributes to mounting pressure on international medical resources and healthcare delivery systems. Epidemiological data from the International Diabetes Federation (IDF) demonstrate that the worldwide prevalence of T2DM among adults aged 20–79 years reached 589 million individuals in 2024, accounting for 11.1% of this demographic cohort. Modeling projections indicate this will increase to 852 million cases by 2050, with the prevalence rate ascending to 13% [2]. In addition, T2DM is one of the leading causes of death in adults globally. Persistent hyperglycemia can trigger various complications such as cardiovascular damage, muscle atrophy [3], and renal impairment. Statistics show that approximately 3.4 million adults aged 20–79 worldwide died from T2DM or its related complications in 2024, accounting for 9.3% of all‐cause deaths in this age group [4]. Diabetic sarcopenia seriously impairs patients′ quality of life and serves as a crucial factor contributing to a series of adverse events including falls, fractures, accelerated progression of T2DM, and mortality [5].

The human anatomical structure encompasses upwards of 600 skeletal muscles, which perform pivotal roles in metabolic homeostasis, motor function, thermal regulation, and visceral organ safeguarding [6]. Sarcopenia is clinically defined as the progressive age‐related attenuation of skeletal muscle mass and functional performance [7]. A multifaceted association exists between T2DM and sarcopenia. Meta‐analytic investigations indicate that diabetic cohorts demonstrate significantly heightened vulnerability to sarcopenia relative to nondiabetic populations [8]. Elderly diabetic patients exhibiting suboptimal glycemic control experience substantially accelerated rates of muscle mass and strength reduction compared with their nondiabetic counterparts [9]. Skeletal muscle constitutes the principal insulin‐responsive tissue, wherein attenuation of muscle mass potentiates IR, impairs glucose utilization efficiency and protein anabolic processes, and ultimately establishes a pathophysiological vicious cycle characterized by reciprocal exacerbation of IR and muscle wasting [10]. The pathogenesis of T2DM‐induced sarcopenia is intricate, which may be associated with skeletal muscle synthesis disorders caused by long‐term poor glycemic control and IR [11]. At present, the management of diabetic sarcopenia mainly relies on controlling the underlying disease itself, including using hypoglycemic agents to regulate blood glucose and modulate protein anabolism [12], dietary nutritional supplementation (e.g., the Mediterranean diet) [13], and resistance exercise training [14]. However, these strategies have the limitation of failing to effectively reverse diabetic sarcopenia. Traditional Chinese medicine (TCM), as a conventional medical system in China, boasts abundant experience in the use of herbal medicines and has achieved favorable therapeutic effects in the treatment of diabetes and sarcopenia. This paper is aimed at reviewing the pathogenesis of T2DM complicated with sarcopenia [15] and the TCM prescriptions for its treatment, laying a foundation for future research on this disease and exploring potential therapeutic drugs that can alleviate T2DM‐induced muscle loss.

### 1.1. Definition of Sarcopenia

Sarcopenia is formally characterized as a disease entity involving progressive attenuation of skeletal muscle mass and functional capacity, exhibiting marked predominance within geriatric populations [16], initially to describe age‐related muscle atrophy; however, the concept did not gain widespread attention from the academic community in the early stage after its proposal. With the continuous deepening of population aging, the critical role of skeletal muscle mass and functional status in the health prognosis of older adults has been increasingly recognized. Through the collaborative efforts of the European Working Group on Sarcopenia in Older People (EWGSOP) and the Asian Working Group for Sarcopenia (AWGS), the conceptual understanding of sarcopenia has undergone continuous refinement. Concurrently, standardized diagnostic criteria have been progressively developed, establishing an essential framework for early detection and screening of this condition.

The EWGSOP promulgated a standardized operational definition in 2010, predicated upon quantitative assessment of muscular mass and motor performance parameters. Sarcopenia was thereby delineated as a clinical syndrome exhibiting the concurrent presence of attenuated muscle mass with either diminished muscular strength or compromised physical functional capacity [17]. The working group emphasized that priority should be given to abnormalities in muscle structure and functional deficits in patients, as the identification of these two aspects constitutes the core component of sarcopenia diagnosis and assessment.

In 2018, EWGSOP reconvened an expert meeting to optimize and revise the diagnostic criteria for sarcopenia, and in 2019, AWGS issued an updated expert consensus. Both authoritative working groups have designated muscle strength as the cornerstone diagnostic criterion for sarcopenia. This recommendation stems primarily from the robust and consistent predictive capacity of muscle strength measurements for adverse health outcomes among older adults [18].

Within the conceptual framework promulgated by EWGSOP and AWGS, a methodical diagnostic protocol for sarcopenia has been articulated, revolving around three pivotal domains: muscular strength, muscle mass, and physical functional capacity. Handgrip strength assessment constitutes the primary modality for evaluating muscular strength, whereas radiographic imaging techniques enable quantification of muscle mass and tissue quality characteristics. Patients manifesting concurrent attenuation of both muscle strength and muscle mass/quality parameters require supplementary evaluation of physical performance metrics.

### 1.2. Operational Diagnostic Methods for Sarcopenia

#### 1.2.1. Muscle Strength Assessment

Grip strength dynamometry constitutes the principal investigative modality for appraising skeletal muscle health and functional integrity, facilitating quantitative determination of isometric contractile capacity within hand and forearm muscular groups. Empirical clinical investigations have substantiated that grip strength parameters exhibit significant correlations with manifold adverse clinical outcomes among older adult cohorts, thereby possessing considerable prognostic assessment significance [19]. Contemporary international standards delineate cutoff parameters for compromised muscular strength at <27 kg for male individuals and <16 kg for female individuals, wherein values beneath these thresholds signify attenuated upper extremity muscle strength [20]. Notwithstanding, grip strength dynamometry exclusively appraises the functional capacity of localized upper limb musculature. Accordingly, integration of the chair stand test becomes imperative for assessing lower extremity muscular strength within geriatric cohorts, thus enabling comprehensive evaluation of systemic skeletal muscle functional status [21].

#### 1.2.2. Muscle Mass Assessment

Skeletal muscle mass quantification constitutes an essential objective parameter for sarcopenia diagnostic determination. Predominant methodological approaches for muscle mass assessment incorporate dual‐energy X‐ray absorptiometry (DEXA), magnetic resonance imaging (MRI), and computed tomography (CT) imaging technologies [22]. These techniques can assess the total body skeletal muscle mass or muscle groups at specific sites, among which CT and MRI are the gold standards for skeletal muscle mass detection due to their higher imaging accuracy [23]. The appendicular skeletal muscle is the main site affected by sarcopenia, and thus appendicular muscle mass is the key observation indicator in the diagnostic process. The ratio of appendicular skeletal muscle mass to height squared (ASM/height^2^) is used as the widely accepted reference indicator in clinical practice.

#### 1.2.3. Physical Function Assessment

Physical function assessment can reflect the overall physical motor capacity of the body, and enables the early identification of occult skeletal muscle functional impairment [24].However, the results of such assessments are susceptible to interference from factors including the test environment, the subjective judgment of the assessor, and the underlying health status of the subject. Therefore, they cannot be used alone as a diagnostic basis for sarcopenia, and need to be comprehensively interpreted in combination with the patient′s overall condition and other objective indicators. The quantification of 4‐m ambulatory speed among older adult cohorts constitutes a comprehensive indicator of physical functional capacity, embodying the synergistic regulatory integration across musculoskeletal, cardiovascular, and neurological systems [25]. Reduced gait velocity serves as an early warning indicator for sarcopenia development and advancement. Additionally, the Short Physical Performance Battery (SPPB) represents another widely utilized clinical evaluation instrument [26]. Developed by the US National Institute on Aging, it is specifically designed for the assessment of lower limb function in older adults, has strong predictive efficacy for long‐term disability outcomes, and can identify early physical function impairment in sarcopenia. It consists of three core tests: the balance test, walking speed test, and chair stand test.

### 1.3. Aging: A Major Risk Factor for Diabetic Sarcopenia

The aging phenomenon constitutes a pivotal independent risk determinant for both diabetes mellitus and sarcopenia, exhibiting well‐characterized causal associations with each pathological entity. Considerable fundamental research evidence has substantiated that aging not only potentiates the disease trajectory of T2DM and sarcopenia, but also serves as the pathophysiological substrate facilitating the emergence of multiple shared risk factors between these two clinical disorders [27].

The hallmark physiological feature of aging is the progressive decline in the function of human organs. Studies have shown that by the age of 80, human skeletal muscle function can decrease by 30%–50% compared with peak adult levels [28]. Such age‐related functional degenerative changes not only directly form the pathological basis of sarcopenia, but also inhibit the replication and proliferation capacity of pancreatic islet *β*‐cells [29], reduce *β*‐cell function and mass, and accelerate the progression of diabetes. Along with advancing age, continuous redistribution of human adipose tissue occurs, with fat progressively shifting from subcutaneous tissue to visceral compartments and forming ectopic lipid deposition in the liver, skeletal muscle and other sites [30]. The persistent accumulation of ectopically deposited intermuscular adipose tissue leads to muscle atrophy, which may be associated with increased local inflammation and impaired muscle contractility [31]. Furthermore, lipids promote the release of inflammatory cytokines to trigger intramuscular inflammation [32], which in turn exacerbates IR and impairs skeletal muscle health and insulin sensitivity.

In the state of aging, an imbalance between the synthetic and catabolic pathways of skeletal muscle proteins leads to muscle mass atrophy and functional impairment. At the microstructural level, this is manifested as a reduction in the number of muscle fibers and decreased contractile function [33]. Meanwhile, aging causes a decrease in the number of skeletal muscle satellite cells, triggering abnormal changes in the quantity and type of muscle fibers. This directly impairs the glucose uptake and metabolism capacity of skeletal muscle and further aggravates glucose metabolism disorders [34]. Irisin, a myokine elaborated by skeletal muscle tissue, exhibits pivotal protective efficacy in the preservation of skeletal muscle mass and functional integrity. This myokine attenuates muscular atrophy via multifaceted mechanistic pathways encompassing: potentiation of satellite cell activation and proliferative capacity, suppression of excessive proteolytic degradation, amelioration of tissue fibrosis and necrotic processes, and enhancement of myocytic membrane structural stability [35]. Meanwhile, Irisin has the capacity to promote the browning transformation of subcutaneous white adipose tissue [36]and enhance systemic insulin sensitivity, thus ameliorating the pathological state of T2DM [37].However, circulating irisin levels decrease significantly with advancing age [38].Low irisin concentration is an independent risk factor for sarcopenia [39], and its insufficient expression further worsens the clinical prognosis of diabetic sarcopenia.

On the basis of reduced protein synthesis capacity, reduced appetite and decreased food intake result in insufficient raw materials for muscle synthesis, which exacerbates the progression of muscle atrophy and aggravates abnormal glucose metabolism. Data show that the daily energy intake of adults aged 40–70 years decreases by an average of 25%. Insufficient energy intake can directly trigger weight loss and skeletal muscle loss, weaken muscle strength and physical motor performance, and simultaneously reduce insulin sensitivity in peripheral tissues [40].

Vitamin D, serving as the metabolic progenitor of 1,25‐dihydroxyvitamin D_3_, represents a pivotal micronutrient regulating systemic glucoregulatory processes and musculoskeletal health [41]. This secosteroid maintains physiological muscle excitability and functional capacity via multifactorial pathways, including modulation of intracellular calcium homeostatic mechanisms and engagement in skeletal muscle stem cell proliferative and differentiative processes [42]. Empirical evidence demonstrates that vitamin D supplementation effectively potentiates muscular strength and attenuates Type II myofiber atrophy [43]. Conversely, vitamin D insufficiency directly impairs insulin biosynthetic and secretory functions, exacerbates peripheral tissue IR, and elevates susceptibility to glucose tolerance abnormalities and T2DM pathogenesis [44].

### 1.4. T2DM and Sarcopenia

Skeletal muscle constitutes a pivotal metabolic organ within the human organism, representing approximately 40% of total body mass and sequestering 50%–75% of whole‐body protein content [45]. This tissue accounts for 30% of basal metabolic rate under resting conditions and surpasses 90% during strenuous physical exertion [46]. As the principal insulin‐responsive organ, skeletal muscle performs an indispensable function in maintaining systemic glucose homeostatic balance [47], mediating the clearance of approximately 80% of circulating glucose from the systemic vasculature [48]. The pathophysiological essence of skeletal muscle atrophy resides in the perturbation of homeostatic balance between protein anabolic and catabolic rates [49]. This syndrome is characterized by attenuated myofibrillar protein content, diminished myofiber population, compromised muscular strength, myofiber atrophic transformation, and reduced fatigue resistance capacity [50]. Skeletal muscle proteolysis is principally mediated through three canonical pathways: the calpain system, the ubiquitin‐proteasome system (UPS), and the autophagy‐lysosome system, which function in a synergistic and complementary fashion during protein degradative processes.

#### 1.4.1. Calpain System

Calpains belong to the papain superfamily, a class of intracellular calcium ion (Ca^2+^)‐dependent cysteine proteases. They are activated upon elevated Ca^2+^ concentration in the sarcoplasm of muscle fibers and mediate the selective hydrolysis of receptor proteins, membrane proteins (such as protein kinase A and protein kinase C [PKC]), cytoskeletal proteins, and contractile proteins. With site‐specific cleavage properties for target proteins, calpains can hydrolyze and release structural proteins of myofibers that are inaccessible to other proteolytic systems, serving as a key initiating step in skeletal muscle protein degradation [51].

#### 1.4.2. UPS

The UPS is the core intracellular system mediating target protein degradation, which breaks down target proteins into amino acid monomers [52]. Through a cascade of reactions catalyzed by a series of cytoplasmic enzymes, the system performs ubiquitination labeling of target proteins, transports the labeled proteins into the proteasome complex for complete degradation, and constitutes the major pathway for the degradation of contractile proteins such as myosin and actin in skeletal muscle [53].

#### 1.4.3. Autophagy‐Lysosome System

This is the third core proteolytic pathway in skeletal muscle, and the only proteolytic system capable of degrading large organelles, protein aggregates, and damaged cellular structures. Under physiological homeostasis, this system exerts a basal quality control function by clearing toxic components including damaged mitochondria and endoplasmic reticulum in cells. Under pathological stress conditions, its excessive activation mediates massive degradation of skeletal muscle structural proteins and accelerates the progression of muscle atrophy [54].

The activation of these three proteolytic machineries is subject to precise modulation via multiple established signaling transduction pathways. Predominant among these are as follows: the interleukin‐6/signal transducer and activator of transcription 3 (IL‐6/STAT3) pathway, the tumor necrosis factor‐*α*/nuclear factor kappa‐B (TNF‐*α*/NF‐*κ*B) pathway, the myostatin/Smad2/3 signaling axis, and the forkhead box protein O1/3 (FoxO1/3) pathway [55].

Skeletal muscle protein anabolic processes are principally modulated through the PI3K‐AKT‐mTOR signaling transduction pathway. Pathway activation is initiated by insulin engagement with plasma membrane receptors [56]. Following insulin‐receptor complex formation, tyrosine kinase activity is potentiated via insulin receptor substrate 1 (IRS‐1), consequentially activating PI3K to catalyze the phosphorylation of phosphatidylinositol 4,5‐bisphosphate (PIP2) into phosphatidylinositol 3,4,5‐trisphosphate (PIP3). PIP3 subsequently mediates the recruitment and activation of 3‐phosphoinositide‐dependent protein kinase 1 (PDK1), which phosphorylates and activates AKT [57]. Ultimately, mTOR is activated through the AKT‐mediated cascade reaction to initiate downstream protein translation and synthesis processes. This pathway is the core regulatory axis for skeletal muscle hypertrophy and muscle mass maintenance.

### 1.5. Pathological Alterations of Muscle Fibers in Diabetic Sarcopenia

Skeletal muscle atrophic conditions are taxonomically categorized into primary and secondary subtypes. Physiological atrophy principally manifests within the context of senescence and attenuated physical activity engagement. Illustratively, among sedentary older adult cohorts, insufficient myofibrillar stimulation precipitates progressive attenuation of muscle mass [58]. Secondary atrophy refers to structural damage and functional decline of skeletal muscle caused by chronic debilitating diseases [59], and T2DM‐related sarcopenia falls into the category of secondary sarcopenia. This type of sarcopenia differs from primary sarcopenia in terms of pathological mechanisms. However, current clinical diagnosis of T2DM‐related sarcopenia still mainly adopts the general operational diagnostic criteria for primary sarcopenia, lacking targeted diagnosis and treatment specifications.

Human skeletal muscle fibers are generally divided into Type I slow‐twitch muscle fibers and Type II fast‐twitch muscle fibers, with distinct differences in structural and functional characteristics between the two types. Type I slow‐twitch fibers have a smaller diameter, abundant mitochondria, and strong oxidative metabolic capacity, relying mainly on oxidative energy supply and being suitable for endurance exercise. Type II fast‐twitch fibers have a larger diameter, sufficient myoprotein and glycogen reserves, and high glycolytic activity, and are mainly involved in explosive‐dominated exercises such as sprinting and weightlifting [60]. There are physiological differences in the distribution ratio of the two types of fibers in different muscle groups of the human body, and this distribution characteristic enables the human body to adapt to the demands of diverse exercise and physiological activities.

There are essential differences in the pathological changes of muscle fibers between primary sarcopenia and diabetic sarcopenia. Primary sarcopenia exhibits as its cardinal pathological feature the atrophic degeneration of Type II myofibers. In distinction, myofiber remodeling in diabetic sarcopenia manifests a characteristic redistribution pattern, encompassing attenuation of Type I oxidative myofiber proportion with concurrent relative augmentation of Type II glycolytic myofiber representation [61]. Case‐control studies comparing patients with obesity and comorbid T2DM against individuals with obesity alone revealed similarly reduced Type I fiber proportions in the T2DM cohort [62]. Detailed pathological examination further demonstrated that glucose transporter Type 4 (GLUT4) density in Type I slow‐twitch fibers of T2DM patients was lower compared with fast‐twitch fibers from the same subjects, and Type I fiber diameter was reduced relative to Type II fast‐twitch fibers [63]. These changes were accompanied by markedly diminished muscle oxidative enzyme activity [64]. Although some investigations failed to detect statistically significant differences in muscle fiber type distribution between T2DM patients and healthy lean or obese controls, they nevertheless observed a trend toward reduced Type I fiber proportions in patients, along with significantly lower muscle oxidative enzyme activity compared with lean controls and elevated intramuscular lipid content [65].

Empirical investigations have substantiated that Type I slow‐twitch myofibers exhibit augmented expression profiles of insulin receptors, hexokinase, glycogen synthase, and GLUT4 protein relative to Type II fast‐twitch myofibers [66], concomitant with enhanced insulin receptor tyrosine kinase activity—suggesting superior insulin sensitivity in Type I slow‐twitch fibers [67]. The diabetic pathological milieu can induce aberrant myofiber phenotypic transitions, manifesting as a shift from Type I slow‐twitch toward Type II fast‐twitch fiber predominance. This phenotypic conversion ultimately culminates in muscle mass attenuation, compromised muscular strength, and diminished glucose uptake capacity [68], thereby potentiating systemic glucoregulatory disturbances and establishing a self‐reinforcing vicious cycle.

### 1.6. Pathological Mechanisms of Diabetic Sarcopenia

**Figure 1:**
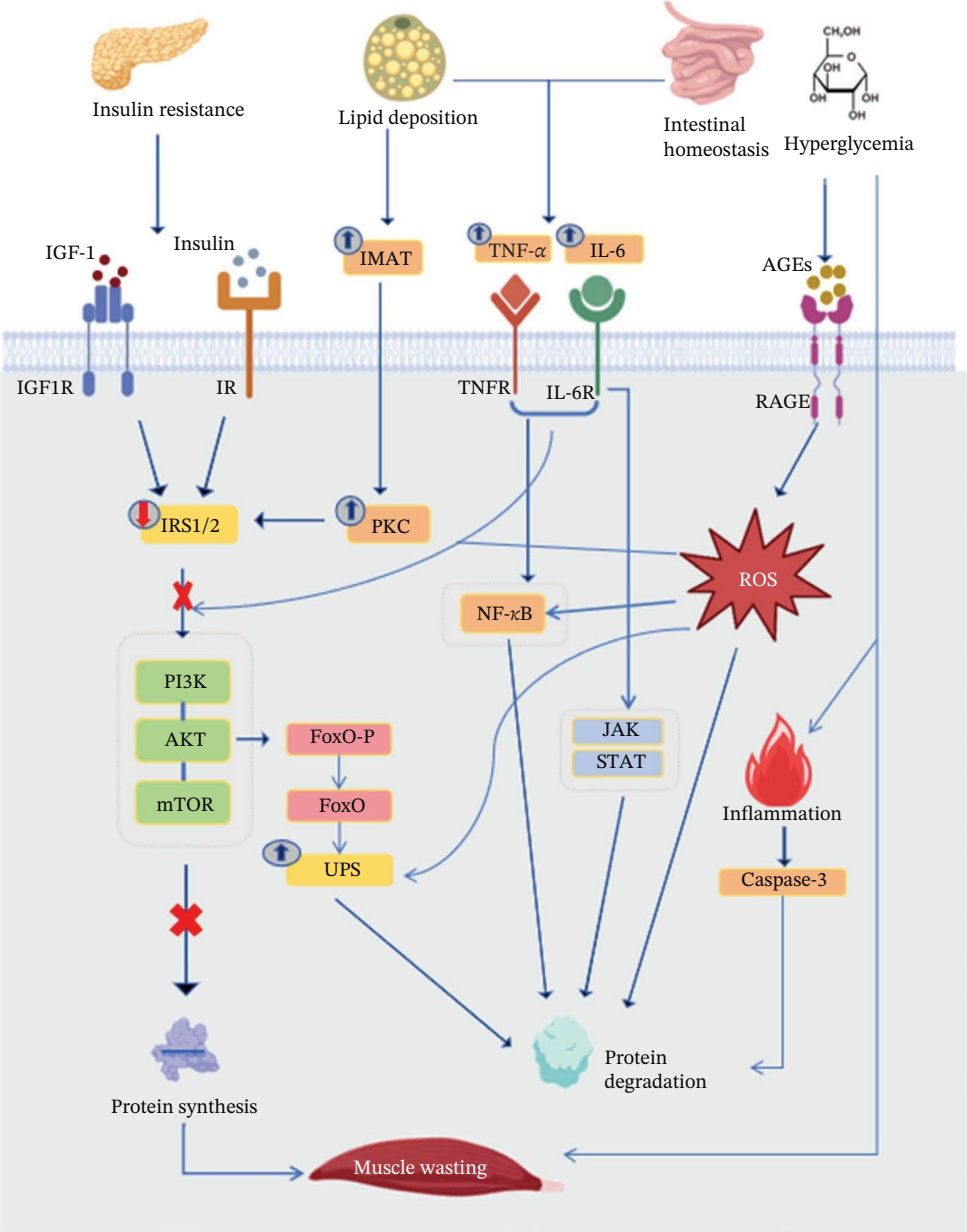
Pathological mechanisms of diabetic sarcopenia.

Accumulating evidence has demonstrated that diabetes mellitus inflicts detrimental effects on skeletal muscle via multiple pathological mechanisms, including IR, hyperglycemia, lipid deposition, oxidative stress, and inflammation (Refer to Figure 1).

#### 1.6.1. IR

IR constitutes the cardinal pathophysiological substrate of T2DM. Insulin performs indispensable functions in metabolic homeostatic regulation. Pathological attenuation of insulin receptor sensitivity and responsiveness within skeletal muscle tissue, concomitant with impaired translocation of GLUT4 to the sarcolemmal surface, culminates in reduced glucose uptake capacity by skeletal muscle [69]. Blood glucose cannot effectively enter muscle tissues to provide energy, leading to fatigue of muscle strength. Long‐term energy deficiency will further exacerbate the decline in muscle strength and affect the repair and synthesis of muscle fibers [70]. In addition, insulin acts as a potent anabolic signal that promotes protein synthesis [71]and muscle growth, as well as inhibits protein degradation [72]. Under homeostatic physiological conditions, insulin‐like growth factor 1 (IGF‐1) mediates anabolic signaling through activation of the PI3K‐Akt‐mTOR transduction cascade, thereby facilitating protein synthesis and muscular hypertrophy, concomitant with suppression of forkhead box O transcription factors (FoxO) to attenuate myofibrillar protein catabolism. In the insulin‐resistant pathological state, however, both insulin and IGF‐1 signaling pathways undergo inhibition, accompanied by attenuated IRS1/2 expression levels [73]. This culminates in obstruction of PI3K‐Akt signal transduction and further suppression of mTOR enzymatic activity [74]. As a canonical regulatory factor governing protein anabolic processes, mTOR functions to initiate protein synthesis and inhibit autophagic degradation. Thus, mTOR inactivation potentiates muscle mass attenuation in patients with T2DM [75].

In physiologically healthy skeletal muscle, the UPS performs essential proteostatic functions through hydrolysis of damaged or misfolded proteins, thereby maintaining myocellular homeostasis. UPS activation is principally mediated through the sequential conjugation of ubiquitin‐activating enzymes (E1), ubiquitin‐conjugating enzymes (E2), and ubiquitin‐protein ligases (E3) to target protein substrates [76]. IR‐induced attenuation of Akt activity precipitates FoxO dephosphorylation and subsequent nuclear translocation. Within the nuclear compartment, FoxO activates transcriptional expression of E3 ubiquitin ligase genes comprising the UPS, resulting in upregulated protein catabolism and accelerated myofibrillar protein depletion [77]and muscle fiber atrophy [78]. Furthermore, WWP1, a constituent member of the E3 ubiquitin ligase family, demonstrates significant association with T2DM‐induced muscle atrophy. Metabolic derangements stemming from IR can potentiate muscle atrophy and compromise muscular strength via the WWP1/KLF15 signaling axis [79].

#### 1.6.2. Hyperglycemia

Hyperglycemia is an independent risk factor for the loss of skeletal muscle mass and function [80]. Under pathophysiological conditions of sustained hyperglycemia, supraphysiological glucose concentrations undergo irreversible covalent conjugation with myofibrillar proteins through nonenzymatic Maillard reaction pathways. This glycation process consequentially culminates in advanced glycation end products (AGEs) sequestration within the skeletal muscle microenvironment [81]. AGEs selectively deposit in fast‐twitch (glycolytic) muscle fibers [82], affecting muscle contraction function by altering protein structure and function. The actin‐myosin cross‐bridge cycling apparatus represents the fundamental molecular substrate of myofibrillar contraction. AGE‐mediated posttranslational modifications induce conformational alterations in both actin and myosin, consequentially attenuating cross‐bridge cycling kinetics and impairing skeletal muscle contractile performance [83]. Long‐term accumulation of AGEs induces progressive cross‐linking and sclerosis of muscle fiber structures, ultimately leading to irreversible deterioration of muscle fiber contractile function, which manifests as reduced muscle strength and impaired exercise capacity [84]. In addition, the receptor for advanced glycation end products (RAGE) is a transmembrane signaling receptor. Binding of AGEs to RAGE increases reactive oxygen species (ROS) in the circulation, triggering muscle cell apoptosis and mitochondrial dysfunction. Simultaneously, it inhibits the PI3K‐Akt signaling pathway, accelerates the degradation of muscle proteins, and ultimately causes skeletal muscle atrophy [85].

#### 1.6.3. Lipid Deposition

Adipose tissue IR attenuates the physiological inhibition of lipolytic activity, consequentially promoting substantial free fatty acid (FFA) efflux into systemic circulation. These FFAs undergo ectopic deposition within multiple insulin‐responsive target organs, encompassing skeletal muscle tissue [86]. Long‐term hyperglycemia can cause disorders of lipid metabolism, further exacerbating the accumulation of intramyocellular adipose tissue (IMAT). Total skeletal muscle mass demonstrates an inverse correlation with adipose tissue mass. Perturbation of body compositional homeostasis directly disrupts glucolipidic metabolic equilibrium, thereby propelling T2DM pathogenesis through a self‐reinforcing vicious cycle. Excessive adipose infiltration into myocellular compartments precipitates elevated concentrations of lipotoxic metabolites, particularly diacylglycerol and ceramide. These metabolites potentiate PKC enzymatic activity, induce serine residue phosphorylation on IRS‐1, and consequentially impair insulin signaling transduction cascades [87]. Adipose tissue itself releases inflammatory factors that induce muscle protein degradation. Adipocytes and macrophages infiltrating adipose tissue produce proinflammatory cytokines, such as interleukin‐6 (IL‐6) and tumor necrosis factor‐*α* (TNF‐*α*) [88], which activate the nuclear factor *κ*B (NF‐*κ*B) pathway [89]to directly induce the expression of enzymes related to intracellular protein degradation, accelerating the loss of skeletal muscle protein.

Adiponectin is a beneficial adipokine in the body that participates in regulating glucose and lipid metabolism and protecting skeletal muscle. Due to excessive fat accumulation and IR, the secretion of adiponectin decreases, resulting in weakened glucose uptake capacity of muscle and insufficient energy supply [90]. Secondly, the inhibitory effect on fat‐induced myofiber damage is lost [91], and the proliferation of muscle satellite cells is hindered, impairing muscle repair capacity [92]. Under normal circumstances, adiponectin alleviates inflammatory responses and antagonizes adipose tissue‐mediated muscle damage by regulating multiple cellular signaling pathways [93]. However, excessive proliferation of adipocytes inhibits adiponectin synthesis, leading to the loss of its aforementioned anti‐inflammatory regulatory function and further exacerbating metabolic disorders and muscle atrophy.

#### 1.6.4. Chronic Inflammation

The caspase superfamily constitutes a group of cysteine proteases characterized by their capacity to cleave substrate proteins at aspartate residues, including the interleukin‐1 (IL‐1) converting enzyme (Caspase‐1) responsible for proinflammatory cytokine maturation [94]. T2DM is a state of chronic inflammation. Hyperglycemia can directly activate inflammatory pathways, induce the activation of Caspase‐3, and subsequently target and cleave myofibrillar proteins in skeletal muscle, accelerating the proteolysis process and disrupting the structural integrity of muscle fibers [95]. Conversely, inflammatory processes suppress protein synthesis through inhibition of the PI3K‐Akt pathway while simultaneously enhancing protein degradation via activation of the UPS. The principal inflammatory mediators participating in this response include TNF‐*α*, IL‐6, and IL‐1 [96]. Augmented TNF‐*α* concentrations within systemic circulation and skeletal muscle tissue precipitate activation of the NF‐*κ*B inflammatory transduction pathway, thereby facilitating myofibrillar atrophy [97] and attenuating myocellular repair capacity [98]. Meanwhile, TNF‐*α* reduces glucose uptake in skeletal muscle by decreasing the expression of GLUT4. Skeletal muscle cells deprived of sufficient energy supply will induce protein breakdown to meet the energy deficit, leading to increased degradation of muscle proteins [99]. The cytokine IL‐6 exerts its catabolic effects through the Janus kinase/signal transducer and activator of transcription (JAK/STAT) signaling pathway. This activation induces expression of genes associated with myocyte apoptosis, culminating in overall skeletal muscle mass reduction [100]and growth inhibition [101]. Of particular concern, persistent IL‐6 elevation disrupts satellite cell functionality and compromises the skeletal muscle′s inherent regenerative capabilities [102].

#### 1.6.5. Oxidative Stress

ROS and reactive nitrogen species (RNS) demonstrate the capability to perturb the structural architecture and functional integrity of proteins, lipids, and nucleic acids. Pathological overaccumulation of ROS and RNS precipitates oxidative stress, consequentially culminating in cellular damage or apoptotic/necrotic cell death [103]. Hyperglycemia promotes the sustained production of ROS by activating the PKC signaling pathway [104]. Pathological ROS overabundance precipitates oxidative damage to essential biological macromolecules within myocytes, including proteins, lipids, and nucleic acids. Oxidatively modified proteins undergo conformational alterations and functional attenuation, consequentially potentiating myocellular apoptotic cascades [105]. Furthermore, ROS ultimately induces muscle atrophy by regulating multiple signaling pathways: It activates the NF‐*κ*B proinflammatory signaling pathway, the UPS, and the autophagy‐lysosome pathway (ALP) to promote muscle protein degradation [106]; meanwhile, it inhibits the Akt‐mTOR pathway, reducing protein synthesis capacity and facilitating muscle atrophy [107].

Excessive ROS impairs the growth and repair capacity of myocytes. Myogenic regulatory factor D (MyoD) is a core regulatory molecule for the differentiation of myoblasts into skeletal muscle fibers [108]. ROS accumulated in skeletal muscle inhibits MyoD expression by downregulating intracellular glutathione levels, impairing myoblast differentiation [109]. In addition, myogenin (MyoG), a constituent member of the MyoD transcription factor family, exhibits continuous involvement throughout the entirety of myogenic processes encompassing growth, development, atrophy, and regeneration. Oxidative stress inhibits MyoG activity, exacerbating muscle atrophy [110]. Myogenic stem cells (MuSCs) are adult stem cells in muscle tissue that are crucial for skeletal muscle development, homeostasis maintenance, and injury repair [111]. However, excessive ROS can significantly inhibit MuSC function, hindering the muscle regeneration process [112].

#### 1.6.6. Dysregulation of Intestinal Homeostasis

The gut microbiota participates in skeletal muscle metabolic homeostasis through metabolite elaboration and intestinal barrier functional regulation. Short‐chain fatty acids (SCFAs) represent canonical metabolites generated through gut microbial fermentation. Empirical investigations have demonstrated that acetate, a constituent SCFA, potentiates muscular endurance capacity in animal models, stimulates peroxisome proliferator‐activated receptor gamma coactivator 1‐alpha (PGC‐1*α*) biosynthesis within skeletal muscle tissue, and augments mitochondrial functional integrity and energetic metabolic processes [113]. Concurrently, SCFAs enhance the metabolic efficiency of skeletal muscle cells through augmentation of mitochondrial activity [114]. Preservation of optimal mitochondrial fatty acid oxidation capacity constitutes a fundamental requirement for skeletal muscle remodeling [115]and prevention of myosteatosis [116]. Supplementary experimental investigations have elucidated that phenolic conjugates elaborated by the gut microbiota demonstrate the capacity to potentiate PI3K signal transduction pathway activity, augment glucose uptake efficiency within muscle fibers, and stimulate myocellular utilization of energetic and metabolic substrates for protein anabolic processes [117]. The aforementioned results indicate that metabolites generated by the gut microbiota can directly or indirectly promote skeletal muscle health.

Diabetic patients generally suffer from imbalances in gut microecological and functional homeostasis, and abnormal intestinal function is a potential factor inducing skeletal muscle damage. In diabetic animal models, the expression of free fatty acid receptor 2 (FFAR2)—a key signaling receptor for SCFAs—is significantly reduced in skeletal muscle. Silencing of FFAR2 blocks the PI3K/AKT/mTOR signaling pathway, which not only decreases muscle formation but also increases oxidative stress‐induced muscle protein degradation [118]. Disruption of intestinal homeostasis impairs intestinal barrier function, leading to the leakage of toxic molecules such as lipopolysaccharides (LPS) into the bloodstream. These toxic molecules can activate macrophages in the body, prompting them to release a large number of proinflammatory cytokines (e.g., TNF‐*α*, IL‐6). When these proinflammatory cytokines reach muscle tissue via the bloodstream, they further activate the NF‐*κ*B and STAT3 pathways, exacerbating muscle protein degradation, inducing muscle fiber atrophy, and reducing muscle mass [119]. Amino acids are core raw materials for muscle protein synthesis, and their deficiency directly hinders the process of skeletal muscle protein synthesis [120]. An imbalanced gut microbiota leads to reduced amino acid synthesis. In addition, animal experiments have demonstrated that under the premise of the same dietary regimen, differences in specific gut microbiota metabolic types result in varying rates of muscle mass growth. This suggests that the gut microbiota can determine muscle growth efficiency by regulating amino acid absorption and muscle anabolic metabolism [121]. On the other hand, the “brain‐gut‐muscle axis” is an important system regulating body energy homeostasis [122]. The gut microbiota can influence signals transmitted by the enteric nervous system (ENS) to the brain through its metabolites, thereby directly regulating appetite [123]. For example, a higher abundance of *Escherichia coli* leads to increased satiety and elevated levels of satiety hormones, preventing overeating or undereating [124]. An imbalanced microbiota may cause abnormal satiety signals, exacerbating insufficient nutrient intake and lack of raw materials for muscle synthesis. Intestinal bacteria not only affect metabolism but also directly produce neurotransmitters such as norepinephrine and dopamine, and regulate the host′s own serotonin synthesis [125]. These neurotransmitters can improve muscle motor function.

### 1.7. Core Pathogenesis of Diabetic Sarcopenia From the Perspective of TCM

In modern academic circles of TCM, diabetes mellitus is generally classified into the category of Xiaoke disease [126]. The core clinical manifestations of sarcopenia are highly consistent with TCM flaccidity syndrome [127], thus sarcopenia is mostly treated in clinical practice following the therapeutic principles of flaccidity syndrome, with definite clinical efficacy [128]. Both diabetes mellitus and sarcopenia belong to diseases of the spleen system in TCM, and their onset and progression are driven by the core factor of abnormal physiological functions of the spleen [129].

According to TCM theory, the core physiological function of the spleen is to transport and transform water and grain essence, namely, to absorb and transfer nutrients from food to nourish the internal zang‐fu organs as well as the limbs and muscles of the body [130]. The water and grain essence transported and transformed by the spleen corresponds to the sum of systemic nutrients in modern medicine, including carbohydrates, proteins, lipids, vitamins, and various trace elements [131]. In addition, TCM theory holds that muscles are subordinate to the spleen system, and the plumpness of muscles is an important external indicator for evaluating the normality of spleen and stomach functions. A healthy and well‐functioning spleen system can generate sufficient energy to nourish and regulate muscle metabolism, thus maintaining plump and strong muscles. In the case of spleen qi deficiency, the failure to transport and transform food into qi, blood, and energy leads to malnutrition of muscles, resulting in pathological manifestations such as limb weakness and motor dysfunction [132].

The core pathogenesis of diabetes mellitus lies in the dysfunction of the spleen in transporting and transforming water and grain essence, which leads to the inability of nutrients such as carbohydrates, proteins, and lipids to be normally metabolized and converted into bioavailable energy for the human body [133]. When the function of the spleen declines, the body reduces the transportation and distribution of water and grain essence to skeletal muscle to prioritize the energy supply of internal organs, which directly impairs the normal growth and functional maintenance of skeletal muscle [134]. Long‐term dysfunction or disorder of the spleen system will cause consumptive manifestations in the body, such as emaciation and skeletal muscle mass loss. Eventually, prolonged Xiaoke disease will be complicated by flaccidity syndrome, which corresponds exactly to diabetes mellitus complicated with sarcopenia [135].

### 1.8. Potential Therapeutic Strategies for Diabetic Sarcopenia: Evidence‐Based Support and Mechanistic Exploration of TCM

TCM possesses an extensive historical legacy in the therapeutic management of diabetes mellitus and sarcopenia. TCM exerts synergistic pharmacological effects via multitarget and multipathway mechanistic networks, thereby facilitating effective intervention in the pathophysiological progression of both disease entities. A systematic review on the clinical application of TCM for diabetes mellitus, which included 160 randomized controlled trials (RCTs), showed that TCM can not only ameliorate oxidative stress, endocrine function, and inflammatory response, but also regulate the AGE pathway, insulin signaling pathway, lipid metabolism pathway, and intestinal microbiota homeostasis, thereby effectively alleviating diabetes mellitus and its related complications [136]. In addition, specific clinical evidence‐based evidence for sarcopenia has also confirmed the definite efficacy of TCM. A dedicated meta‐analysis focusing on Chinese herbal medicine for the treatment of sarcopenia, which enrolled a total of 1440 subjects from 17 relevant RCTs, confirmed that Chinese herbal medicine can significantly increase the total effective rate of sarcopenia treatment. The study further performed stratified analysis on the three core outcome measures of sarcopenia diagnosis and treatment: muscle mass, muscle strength, and physical function. Its core findings explicitly support that Chinese herbal medicine can serve as a complementary and alternative treatment modality for the prevention and treatment of sarcopenia, with definite clinical benefits [137]. Specifically, in terms of muscle mass, Chinese herbal medicine significantly increased the appendicular skeletal muscle mass index (ASMI) of patients; in terms of muscle strength, it markedly improved grip strength and peak knee extension torque, with a more prominent improvement effect on muscle strength in elderly patients over 70 years old; in terms of physical function, it significantly elevated the 6‐m walking speed and SPPB score of patients [138]. At the preclinical research level, active components of Chinese herbal medicine exert beneficial antisarcopenia effects by improving protein metabolism, inhibiting inflammation and oxidative stress, ameliorating IR, and regulating mitochondrial function [139]. These mechanisms are highly consistent with the core action pathways of TCM intervention in diabetes mellitus, further suggesting the therapeutic potential of TCM for diabetic sarcopenia.

Diabetic sarcopenia, recognized as a chronic complication of diabetes mellitus, has emerged as a distinct clinical entity in recent years. Currently, clinical practice lacks standardized therapeutic protocols specifically targeting this condition, and high‐quality evidence‐based research on TCM interventions remains limited. Existing efficacy evidence is mostly concentrated in preclinical studies, and numerous animal experiments have confirmed that TCM intervention has the potential to alleviate diabetic sarcopenia. Although the overall evidence is still in the preliminary stage, it has shown clear clinical application value and research prospects. Meta‐analytic investigations specifically directed at diabetic sarcopenia have furnished supplementary evidence‐based documentation. Empirical data substantiate that TCM exerts significant therapeutic effects on the cardinal pathophysiological features of diabetic sarcopenia, encompassing: augmentation of gastrocnemius muscle mass, expansion of myofiber cross‐sectional area (CSA), amelioration of muscle tissue pathological morphology, and potentiation of grip strength in patient populations. The results of these analyses also confirmed that TCM treatment has a significant improvement effect on hypoglycemia‐related indicators in patients [140].

TCM possesses the intervention advantage of multitarget and multipathway regulation [141], and can directly modulate the homeostasis of skeletal muscle protein turnover. Numerous animal studies have found that TCM treatment for diabetic sarcopenia can promote skeletal muscle protein synthesis by activating the PI3K‐AKT‐mTOR signaling pathway, while inhibiting protein degradation pathways, thereby maintaining the metabolic balance of skeletal muscle proteins [142]. IR, hyperglycemia, lipid deposition, chronic inflammation, oxidative stress, and mitochondrial dysfunction are the core pathological links that mediate the imbalance of skeletal muscle protein homeostasis and ultimately induce diabetic sarcopenia. By alleviating IR [143], exerting anti‐inflammatory and antioxidative effects [144], and enhancing mitochondrial function [145], Chinese herbal medicine can ameliorate multiple core pathological links of diabetic sarcopenia and exert a systemic intervention effect on the disease.

#### 1.8.1. TCM Compound Prescriptions and Chinese Patent Medicines (Refer to Table 1)

**Table 1:** TCM Compound prescriptions for diabetic sarcopenia: clinical and preclinical evidence.

TCM	Application	Clinical efficacy/mechanism study	Refs.
Sijunzi decoction	100 Elderly sarcopenia patients	Postintervention, grip strength, ASMI and SPPB scores were elevated in both groups, with significantly higher values in the treatment group versus the control group (*p* < 0.05). Postintervention, serum ALB, PA ,and TP levels were elevated in both groups, with significantly higher levels in the treatment group versus the control group (*p* < 0.05). The total effective rate was 92.00% (46/50) in the treatment group and 75.51% (37/49) in the control group(*p* < 0.05).	Wang Q. [146]
	Clinical sample comprising 100 geriatric cases of Type 2 diabetes mellitus	All blood glucose‐related biochemical parameters decreased significantly in both groups (*p* < 0.05), with the treatment group showing significantly lower levels than the control group (*p* < 0.05). The treatment group had higher HOMA‐*β*, lower HOMA‐IR, lower hs‐CRP, TNF‐*α*, MDA and nesfatin‐1, and higher SOD and SHBG than the control group (all *p* < 0.05).	Zhang J. [147]
	40 SD rats	The treatment group showed significant increases in limb grip strength, QF wet weight, myosin content, CSA, myonucleus count, and the number of Pax7 and MyoD positive cells. The mechanism underlying these effects is related to the activation of MuSC proliferation and differentiation, which in turn increases myonuclei number, expands CSA, boosts muscle strength, and improves muscle atrophy.	Ma D. [148]
	40 SD rats	Sijunzi decoction promoted insulin and GIP secretion and inhibited glucagon and gastrin secretion. Meanwhile, GLP‐1 level was significantly correlated with blood glucose, insulin, glucagon, and other indicators (*p* < 0.05).	Zhou L.J. [149]
Buzhong Yiqi decoction	40 sarcopenia patients aged ≥60 years	Both groups showed reduced IL‐6 and TNF‐*α* levels, with lower levels in the treatment group (*p* > 0.05). Buzhong Yiqi decoction effectively prevents and treats sarcopenia by inhibiting inflammation.	Chen Y.Y. [150]
	80 T2DM patients complicated with sarcopenia	Posttreatment, gait speed, grip strength, ASM, and ASMI were significantly elevated in the treatment group versus baseline (*p* < 0.05), and were significantly higher in the treatment group than in the control group (*p* < 0.05). Meanwhile, IL‐6, TNF‐*α*, and CAF levels in the treatment group were significantly decreased after treatment (*p* < 0.05).	Wang Q. [151]
	35 SD rats	Compared with the control group, the treatment group had significantly increased rat body weight, grip strength, and exhaustive swimming time (*p* < 0.01), with markedly ameliorated gastrocnemius histopathology. The AMP/ATP ratio was significantly decreased, Atrogin‐1 and MuRF‐1 expression was significantly downregulated (*p* < 0.01), MyoD and myogenin expression was significantly upregulated (*p* < 0.01), and the protein levels of p‐AMPK/AMPK, SIRT1, and PGC‐1*α* were significantly increased (*p* < 0.01).	Wang Y. [152]
	40 SD rats	Compared with the control group, the treatment group showed significantly increased forelimb grip strength, muscle wet weight/body weight ratio, citrate synthase, Ca^2+^‐ATPase and muscle glycogen levels, with markedly improved muscle and mitochondrial microstructure (*p* < 0.05). Cleaved‐caspase 3 expression was significantly decreased in the treatment group (*p* < 0.05). The expression of PI3K, p‐Akt, Akt, leptin, and IGF‐1 was significantly upregulated, whereas MUSCLIN, p‐GSK‐3*β*, HK‐2, p‐p65, TRAF6, IL‐6, and TNF‐*α* were significantly downregulated in the treatment group (all *p* < 0.05).	Fan F.X. [153]
Bazhen decoction	118 Sarcopenia patients	Posttreatment, clinical symptoms and exercise tolerance indicators were significantly improved in both groups (*p* < 0.05), with a significantly longer 6MWD in the treatment group versus the control group (*p* < 0.05). The treatment group showed significantly improved muscle health indicators (*p* < 0.05), with significantly higher levels of the three core muscle health metrics (muscle function, strength and mass) compared with the control group (*p* < 0.05).	Zhou J. [154]
	100 T2DM patients	Posttreatment, blood glucose level and BMI in the treatment group were decreased, with significantly lower levels versus the control group (*p* < 0.05).	Lian H.B. [155]
Shenling Baizhu San	140 T2DM patients complicated with sarcopenia	Postintervention, the intervention group had significantly lower HbA1c and adverse reaction incidence, and significantly higher grip strength and 6‐m gait speed versus the control group (all *p* < 0.05).	Jiang H.H. [156]

1. Sijunzi decoction, a classical prescription documented in the Song Dynasty pharmaceutical compendium Formulas of the Peaceful Benevolent Dispensary, encompasses four medicinal constituents: Ginseng Radix et Rhizoma, Atractylodis Macrocephalae Rhizoma, Poria Cocos, and Glycyrrhizae Radix et Rhizoma. This pharmacological preparation is taxonomically classified within the tonic therapeutic category of TCM. Its cardinal therapeutic indications encompass qi supplementation and splenic function potentiation. As a paradigmatic formula for the management of spleen‐stomach qi deficiency syndrome, it demonstrates extensive clinical applicability. A clinical trial encompassing 100 geriatric patients with sarcopenia comorbid with spleen‐stomach qi deficiency syndrome substantiated that modified Sijunzi decoction adjunctively administered with resistance band training elicited superior therapeutic outcomes relative to conventional intervention alone. Multidimensional improvements encompassed muscle mass augmentation, muscle function enhancement, physical mobility optimization, nutritional status amelioration, and attenuation of spleen‐stomach qi deficiency symptomatology. These findings validate the combined therapeutic regimen as an efficacious intervention strategy for this clinical condition [146]. Meanwhile, Sijunzi decoction has been proven effective in the treatment of diabetes mellitus. A clinical observation on the combination of Sijunzi decoction and Saxagliptin for T2DM showed that the addition of Sijunzi decoction could further reduce blood glucose levels and improve IR [147]. Animal experiments have demonstrated that Sijunzi decoction can activate the MuSC/MND pathway to prevent disuse atrophy [148]. The mechanistic basis underlying amelioration of glucose metabolism may be attributable to modulation of gastrointestinal‐islet axis functional integrity. Through concomitant inhibition of glucagon secretory activity and potentiation of insulin release, this therapeutic intervention elicits significant attenuation of fasting blood glucose levels in rodent models [149].
2. Buzhong Yiqi decoction, a classical prescription first enumerated in Li Dongyuan′s Jin‐Yuan Dynasty pharmaceutical treatise on the spleen and stomach, encompasses eight medicinal constituents: Astragali Radix et Rhizoma, Atractylodis Macrocephalae Rhizoma, Ginseng Radix et Rhizoma, Angelicae Sinensis Radix, Cimicifugae Rhizoma, Bupleuri Radix, Citri Reticulatae Pericarpium, and Glycyrrhizae Radix et Rhizoma. Its cardinal therapeutic indications per TCM theory encompass supplementation of middle jiao qi and elevation of yang qi for the management of qi sinking patterns. Through modulation of spleen–stomach functional homeostasis, this formula potentiates “middle qi” levels, thereby facilitating enhancement of muscular functional capacity. In the therapeutic management of geriatric sarcopenia, Buzhong Yiqi decoction elicits significant attenuation of circulating inflammatory cytokine concentrations, notably IL‐6 and TNF‐*α*. This immunomodulatory effect consequentially ameliorates the inflammatory cytokine‐induced potentiation of myofibrillar protein catabolic processes [150]. For patients with T2DM complicating sarcopenia, the decoction can effectively improve their muscle strength [151]. Preclinical investigations in animal models have substantiated that Buzhong Yiqi decoction mediates its pharmacological effects through two canonical signaling cascades: (1) Potentiation of AMPK/SIRT1/PGC‐1*α* signal transduction, culminating in enhanced exercise capacity and myofibrillar mass augmentation in 5‐fluorouracil (5‐FU)‐induced sarcopenia murine models; [152] (2) attenuation of TRAF6/NF‐*κ*B pathway activity, resulting in diminished inflammatory cytokine elaboration. Concomitantly, this intervention modulates LEPTIN, MUSCLIN, and IGF‐1 concentrations, restores physiological PI3K/Akt pathway activation, potentiates myocellular glucose transport and glycogen synthetic processes, and consequentially ameliorates skeletal muscle functional integrity [153].
3. Bazhen decoction, a classical octuple herbal prescription, encompasses the following medicinal constituents: Ginseng Radix et Rhizoma, Atractylodis Macrocephalae Rhizoma, Poria Cocos, Glycyrrhizae Radix et Rhizoma, Rehmanniae Radix Preparata, Angelicae Sinensis Radix, Chuanxiong Rhizoma, and Paeoniae Radix Alba, is traditionally used for syndrome of deficiency of both qi and blood, and is commonly applied for antisenescence in the elderly population [157]. Recent clinical studies have confirmed that Bazhen decoction can effectively improve muscle motor performance in patients with age‐related sarcopenia [154]. Another RCT involving 146 patients with T2DM showed that Bazhen decoction could significantly reduce patients′ blood glucose levels [155]. Bazhen decoction can significantly reduce patients′ blood glucose levels, highlighting its potential application value in the treatment of diabetic sarcopenia.
4. Shenling Baizhu San, recorded in Formulas of the Peaceful Benevolent Dispensary, is composed of 10 Chinese medicinal herbs including Ginseng Radix et Rhizoma, Atractylodis Macrocephalae Rhizoma, Poria Cocos, Dioscoreae Rhizoma, Nelumbinis Semen, Dolichoris Lablab Semen, Coicis Semen, Amomi Fructus Rotundus, Platycodonis Radix, and Glycyrrhizae Radix et Rhizoma. It possesses the effects of invigorating the spleen and replenishing qi, as well as promoting diuresis to relieve diarrhea. Modern studies have confirmed that it exerts multiple pharmacological actions, such as alleviating inflammatory responses, antagonizing oxidative stress, and regulating intestinal flora [158]. A RCT involving 140 patients with T2DM complicated by sarcopenia showed that after 3 months of intervention, the treatment group, receiving Shenling Baizhu San combined with conventional treatment, had a significantly greater reduction in glycated hemoglobin and a more pronounced improvement in muscle motor performance compared with the control group [156].

#### 1.8.2. TCM Active Components (Refer to Table 2)

**Table 2:** Bioactive components of single Chinese herbs for diabetic sarcopenia: targets and mechanisms.

TCM	Bioactive components	Model	Target/signal pathways	Cellular effects	Ref.
Astragali Radix et Rhizoma	Astragalus polysaccharides (APS)	SD rats	PI3K‐AKT‐FOXO1 signal pathways, IL‐6, TP53, HSF1, and MMP‐9	Restoration of myocyte morphology, recovery of the balance between protein synthesis and degradation, inhibition of myocyte apoptosis, and protection of the extracellular matrix.	Kong Y.H. [159]
Puerariae Lobatae Radix	Puerarin	SD rats	Akt/mTOR signal pathways, LC3/p62 signaling pathway, Atrogin‐1, and Murf‐1	Inhibition of protein degradation and promotion of protein synthesis, suppression of inflammation, amelioration of insulin resistance, and inhibition of myocyte apoptosis.	Yin L. [160]
Lycii Fructus	*Lycium barbarum* extract	C57BL/6 J mice	AMPK/SIRT1/PGC1*α* signal pathways	Inhibitory effect on myotube atrophy, promotive effect on myogenic differentiation, ameliorative effect on mitochondrial energy metabolism.	Zhou X. [161]
Salviae Miltiorrhizae Radix et Rhizoma	Magnesium tanshinonate	C57BL/6 J mice	PI3K‐AKT‐FOXO1 signal pathways, PI3K/AKT/mTOR signal pathways, MAFbx/Atrogin‐1, and MuRF‐1	Inhibition of muscle protein degradation and activation of muscle protein synthesis.	Cheng T.L. [162]
Codonopsis Radix	Codonopsis saponin I	C57BL/6 J mice	PI3K/AKT/mTOR signal pathways, SIRT1/PGC‐1*α* signal pathways, MuRF1, Atrogin‐1, SIRT1, and PGC‐1*α*	Reversal of myotube atrophy, promotion of muscle protein synthesis, inhibition of muscle protein degradation, and improvement of energy metabolic status in myocytes.	Kim T.Y. [163].
	Codonopsis polysaccharides	C57BL/6 J mice	Nrf2 signaling pathway, SOD, and CAT	Repair of skeletal muscle insulin signaling pathway, alleviation of cellular oxidative damage, and enhancement of cellular antioxidant capacity.	Zhang Y. [164]
Scutellariae Radix	Baicalin	C2C12 cell	Cell death pathway, ROS, MDA, Caspase‐3, and caspase‐9	Protection of myoblast viability, inhibition of myoblast apoptosis, scavenging of excessive reactive oxygen species (ROS), and preservation of mitochondrial structure and function.	Pan Y. [165]

1. Astragali Radix et Rhizoma, a traditional Chinese medicinal material, is botanically defined as the dried root system of *Astragalus membranaceus* (Fisch.) Bunge var. mongholicus (Bunge) P.K. Hsiao or *Astragalus membranaceus* (Fisch.) Bunge, taxonomically classified under Fabaceae, has traditional effects of replenishing qi and lifting yang, as well as consolidating the exterior to arrest sweating. Contemporary pharmacological evidence has elucidated that this botanical medicine manifests pleiotropic pharmacological effects, encompassing: antisenescence, antidiabetic, anti‐inflammatory, antioxidant, antiinfective, cytoprotective, and immunomodulatory activities [166]. Astragalus polysaccharides (APS), the core active component of Astragali Radix et Rhizoma, can enhance skeletal muscle glucose utilization and exert a protective effect on skeletal muscle [167]. Animal experiments have shown that after administration of APS to diabetic rats, the volume of gastrocnemius muscle cells increased and the levels of diabetic protective factors elevated. The underlying mechanism potentially involves modulation of the PI3K‐AKT‐FOXO1 signaling pathway. This includes reduction of inflammatory factor levels, specifically IL‐6 and TP53, along with regulation of HSF1 and MMP‐9 activity, ultimately leading to amelioration of muscle atrophy in rats with diabetic sarcopenia [159].
2. Puerariae Lobatae Radix, with a medicinal history of over 1000 years in China, has been confirmed by modern pharmacology to possess multiple pharmacological effects, including alleviating alcoholism, hepatoprotection, antitumor, anti‐inflammatory, and antioxidant activities [168]. As the principal bioactive monomer derived from Puerariae Lobatae Radix, puerarin demonstrates substantial inhibitory activity against skeletal muscle atrophy. Its mechanism of action involves activation of the Akt/mTOR signaling cascade to enhance protein synthesis, coupled with suppression of the autophagic pathway to diminish protein degradation. In streptozotocin (STZ)‐induced diabetes mellitus rat models, puerarin can significantly alleviate skeletal muscle atrophy [160].
3. Lycii Fructus is botanically defined as the dried ripe fruit of *Lycium barbarum* L., taxonomically categorized under the Solanaceae family, and is traditionally used for replenishing the liver and kidney, as well as benefiting essence to improve eyesight. Recent studies have focused on nanovesicles derived from *Lycium barbarum* extract, which exhibit unique advantages in resisting skeletal muscle atrophy and aging. These pharmacologically active components exert antiatrophic effects against dexamethasone (DEX)‐induced skeletal muscle atrophy via potentiation of the AMPK/SIRT1/PGC‐1*α* signal transduction pathway. Concomitantly, they modulate myocellular energy metabolic processes, including amino sugar/nucleotide sugar metabolism and oxidative phosphorylation, while facilitating myogenic regenerative processes [161]. In addition, retrospective studies have confirmed that Lycii Fructus and its various active components regulate blood glucose through multiple pathways [169], thereby providing a theoretical basis for their therapeutic application in diabetic sarcopenia.
4. Magnesium tanshinonate, the predominant bioactive monomeric constituent derived from Salviae Miltiorrhizae Radix et Rhizoma, manifests protective efficacy against skeletal muscle pathology while exerting regulatory effects on metabolic homeostasis. This compound inhibits muscle protein degradation through suppression of muscle‐specific ubiquitin E3 ligases, while simultaneously activating the PI3K‐Akt‐FoxO1 signaling pathway to enhance protein synthesis. Additionally, magnesium tanshinonate attenuates inflammation‐mediated muscle atrophy by suppressing the TNF‐*α*/TNFR1/NF‐*κ*B signaling cascade. Furthermore, it ameliorates high‐fat diet‐induced metabolic disturbances, including IR, thereby conferring dual protective benefits on skeletal muscle and improving disorders of glucose and lipid metabolism [162].
5. Codonopsis Radix has traditional effects of replenishing middle qi and lifting yang, as well as invigorating the spleen and benefiting the lung. Modern studies have confirmed that it intervenes in various diseases through mechanisms such as inflammation regulation, oxidative stress modulation, immune regulation, and apoptosis inhibition [170]. Research has shown that Codonopsis Radix and its active component, codonopsis saponin I, can promote skeletal muscle protein synthesis while reducing muscle protein degradation by regulating and activating the PI3K/Akt/mTORC1 pathway. Additionally, it up regulates the SIRT1/PGC‐1*α* pathway to enhance mitochondrial biogenesis, improve skeletal muscle energy metabolism, and cell viability, thereby further alleviating muscle atrophy [163]. Meanwhile, Codonopsis Radix can inhibit lipid accumulation in skeletal muscle by repairing the impaired PI3K/Akt pathway and regulating lipid metabolism, thus preventing muscle atrophy [171]. Codonopsis polysaccharides (CPS), as the main active component, can effectively alleviate high‐fat and high‐sugar diet‐induced IR in mice and control blood glucose levels by activating antioxidant signaling pathways [164].
6. Scutellariae Radix: The main components of Scutellariae Radix are flavonoids, among which baicalin is the most abundant. In‐depth studies on baicalin in recent years have revealed that it can phosphorylate IRS‐1, enhance the function of GLUT‐4, and simultaneously regulate three signaling pathways—AMPK, PI3K/Akt, and MAPK/ERK—to improve glucose uptake capacity and regulate blood glucose levels [172]. In terms of skeletal muscle protection, studies have indicated that baicalin can alleviate oxidative stress, improve mitochondrial dysfunction, and inhibit the mitochondria‐mediated apoptotic pathway to suppress the apoptosis of C2C12 myoblasts. Meanwhile, it exerts anti‐inflammatory effects, repairs the metabolism and structure of damaged skeletal muscle, and thus holds potential for application in scenarios involving skeletal muscle injury mediated by oxidative stress and apoptosis—a pathological feature highly relevant to diabetic sarcopenia [165].

## 2. Conclusion

Concomitant with the global demographic transition toward population aging, the epidemiological burden of diabetic sarcopenia has demonstrated a persistent upward trajectory. This pathological entity has emerged as a clinically significant complication of diabetes mellitus, exerting profound detrimental effects on patient quality of life metrics while concurrently elevating the incidence of adverse health outcomes. The pathogenic architecture of this clinical entity is characterized by multifactorial complexity, encompassing diverse pathological cascades including: IR, hyperglycemia, lipid deposition, chronic inflammation, oxidative stress, and intestinal homeostatic dysregulation. These mechanistic pathways exhibit bidirectional interactions, culminating in a self‐amplifying pathophysiological cycle that synergistically precipitates skeletal muscle mass attenuation and functional performance decline. Although modern medicine intervenes through methods such as hypoglycemic agents, nutritional support, and resistance exercise training, it is difficult to effectively reverse the core pathological process of muscle damage.

Based on extensive clinical practice, TCM has demonstrated the overall regulatory advantage of “co‐treatment of glucose metabolism and muscle function” in the treatment of diabetic sarcopenia. Classic TCM compound formulas, including Sijunzi decoction, Buzhong Yiqi decoction, Bazhen decoction, and Shenling Baizhu powder, employ therapeutic principles of qi supplementation and spleen fortification, as well as dual tonification of qi and blood. These prescriptions demonstrate multifaceted therapeutic effects: they regulate glucose and lipid metabolic homeostasis, enhance insulin sensitivity, stimulate muscle protein synthesis, and suppress protein catabolism through modulation of key signaling cascades including PI3K‐Akt‐mTOR and AMPK/SIRT1/PGC‐1*α* pathways. Meanwhile, they alleviate inflammatory responses and oxidative stress, and repair the balance of intestinal flora.

The active ingredients of single Chinese medicines also play an important role. Astragalus polysaccharide, puerarin, *Lycium barbarum* extract, magnesium tanshinonate, and so on achieve the protection of skeletal muscle and the improvement of metabolic disorders by targeting inflammatory factors (IL‐6, TNF‐*α*), oxidative stress pathways, the UPS, and so on. Their multitargeted action characteristics are highly consistent with the complex pathological mechanism of diabetic sarcopenia.

TCM, distinguished by its pharmacological attributes of multicomponent composition, multitarget mechanistic networks, and holistic regulatory paradigms, has manifested distinctive therapeutic efficacy in the management of chronic complex pathological entities, notably diabetic sarcopenia. The foundation of its therapeutic efficacy lies in the synergistic effects of compound compatibility. Notwithstanding the aforementioned progress, the extant research landscape remains constrained by substantial methodological deficiencies that necessitate critical consideration. Most modern studies adopt a single‐target and single‐pathway verification mode, which is difficult to comprehensively elucidate the network regulatory mechanism of hundreds to thousands of components in TCM compounds. On this basis, scholars have proposed to decipher the synergistic mechanism of TCM compounds from the holistic level of biological systems, replacing single‐target and single‐pathway verification with integrated multiomics technology, to uncover the holistic network regulatory mechanism of TCM from the dimensions of pharmaceutical ingredients, therapeutic targets, and action pathways.

In summary, through the synergistic intervention of compound prescriptions and active ingredients of single medicines, TCM has significant advantages in improving glucose metabolism, protecting skeletal muscle function, and breaking the vicious pathological cycle in patients with diabetic sarcopenia. This scholarly work systematically delineates the pathophysiological architecture of diabetic sarcopenia alongside the mechanistic targets of TCM therapeutic interventions, thereby furnishing a theoretical substrate and clinical reference framework for preventive and therapeutic modalities. Prospective research imperatives encompass execution of methodologically rigorous, high‐quality clinical investigations to elucidate precise molecular target profiles and pharmacokinetic dose‐response relationships of TCM interventions, optimization of multicomponent herbal compatibility protocols, and advancement of standardized, precision‐oriented TCM therapeutic applications in diabetic sarcopenia management.

## Author Contributions

Zicheng Ye and Haoyu Yuan contributed equally to this work and are co‐first authors. They jointly developed the search strategy, screened literature, and drafted the manuscript. Hanyu He took part in literature screening and manuscript revision. As the corresponding author. Weibo Wen conceived the study design and polished and revised the full manuscript.

## Funding

This study was supported by the Yunling Scholar Special Project of Yunnan "Xingdian Talent Support Program" (Grant No. XDYC‐YLXZ‐2022‐0027).

## Disclosure

All authors have read and approved the final version of the manuscript. Weibo Wen had full access to all of the data in this study and takes complete responsibility for the integrity of the data and the accuracy of the data analysis.

## Conflicts of Interest

The authors declare no conflicts of interest.

## Data Availability

All data discussed in this review are derived from previously published studies and can be accessed from the cited references.
